# Improving Localization Accuracy: Successive Measurements Error Modeling

**DOI:** 10.3390/s150715540

**Published:** 2015-07-01

**Authors:** Najah Abu Ali, Mervat Abu-Elkheir

**Affiliations:** 1 College of Information Technology, United Arab Emirates University, Al-Ain 15551, Abu Dhabi; 2 Faculty of Computer and Information Sciences, Mansoura University, Mansoura 35516, Egypt; E-Mail: mfahmy78@mans.edu.eg

**Keywords:** localization, Gauss–Markov model, location prediction

## Abstract

Vehicle self-localization is an essential requirement for many of the safety applications envisioned for vehicular networks. The mathematical models used in current vehicular localization schemes focus on modeling the localization error itself, and overlook the potential correlation between successive localization measurement errors. In this paper, we first investigate the existence of correlation between successive positioning measurements, and then incorporate this correlation into the modeling positioning error. We use the Yule Walker equations to determine the degree of correlation between a vehicle’s future position and its past positions, and then propose a *p*-order Gauss–Markov model to predict the future position of a vehicle from its past *p* positions. We investigate the existence of correlation for two datasets representing the mobility traces of two vehicles over a period of time. We prove the existence of correlation between successive measurements in the two datasets, and show that the time correlation between measurements can have a value up to four minutes. Through simulations, we validate the robustness of our model and show that it is possible to use the first-order Gauss–Markov model, which has the least complexity, and still maintain an accurate estimation of a vehicle’s future location over time using only its current position. Our model can assist in providing better modeling of positioning errors and can be used as a prediction tool to improve the performance of classical localization algorithms such as the Kalman filter.

## 1. Introduction

Vehicle self-localization is an essential requirement for many of the safety applications envisioned for vehicular networks. However, to achieve a high degree of localization accuracy, computations that involve arrays of sensors and incorporate the physical characteristics of wireless communication channels may increase the complexity of the localization system. Accurate and efficient vehicle localization mechanisms are essential to the adoption of emerging location-based applications. Global Navigation Satellite Systems such as the Global Positioning System (GPS) have been widely used to provide localization for mobile nodes. However, it has been established that these systems may not always provide dependable position information, due mostly to signal deterioration in urban environments [1,2]; contexts in which emerging vehicular applications increasingly require accurate localization. 

Research in localization accuracy is focused on the incorporation of additional data sources in the localization process in order to enhance the accuracy of a vehicle’s GPS measurements. Example localization techniques are the fusion of data from other sensors [2], the use of anchor landmarks and maps [3], and cooperative positioning, the verification and correction of location data among neighbor nodes [4,5]. However, deploying these methods may incur additional costs and increase the complexity of the localization scheme.

Current localization schemes focus on modeling the localization error itself. This is translated to current literature being interested mainly in calculating the current location of vehicles. Little or no work considers time correlation between successive positioning errors in order to account for future positioning. The Kalman filter is a commonly used filtering technique for estimating vehicle positions, but it needs to be parameterized by the measurement noise, which is unknown and is usually modeled as a random variable with Gaussian distribution. Practical implementation of the Kalman Filter is often difficult due to the inability of getting a good estimate of the noise covariance matrices.

Motivated by the above observations, we first investigate the existence of correlation between successive measurements, and determine the degree of correlation if it does exist. We proceed to present a mathematical model for positioning errors that takes into consideration the time correlation. Since vehicles exhibit a predictable and constrained behavior in terms of mobility, we anticipate that the future position of a vehicle will be correlated with its previous positions. Verifying this hypothesis via our proposed model can help improve the localization accuracy without incurring additional costs in terms of complexity. We propose to model the successive positioning errors as a *p*-order Gauss–Markov model, and investigate the order *p* in the *p*-order Gauss–Markov model over four datasets and verify the existence of time correlation. The proposed model can therefore be used as a prediction tool for other localization methods, and can be used for localization whenever the other methods fall short. The main benefit of this approach is that it produces vehicle positioning with a high level of accuracy with no need for fusion of additional data, making the localization process self-contained within the vehicle. By integrating our model into the Kalman filter, we show that the rigorous modeling of positioning errors can assist in designing robust algorithms that predict the future locations of mobile node and improve the accuracy of localization.

The contribution of this paper is three-fold; we establish the time correlation of successive location measurement errors, we define a *p*-order Gauss–Markov model that can be used for the prediction of future location while taking positioning error into account, and we use this model to enhance the accuracy of the prediction step in existing Kalman filter implementations with no need for data fusion. The proposed model can be applied to any localization method, given that the consecutive measurements are within the time correlation epoch. 

The rest of this paper is organized as follows: Section 2 discusses current approaches to vehicle localization. Section 3 describes the *p*-order Gauss–Markov model used for localization. Section 4 highlights the potential applications of our proposed model. In Section 5, we outline the simulation setup used to verify the proposed model, and discuss the results. Finally, Section 6 concludes the paper.

## 2. Related Work

Current work that is focused on improving measurement accuracy for vehicular networks follows four main directions: employing new tools to enhance accuracy, such as machine learning and cooperative techniques; data fusion of different sources of information; attempts to provide accurate empirical; and theoretical modeling for measurement errors; and measurement uncertainty benchmarking and evaluation. We briefly discuss the efforts made in each of those directions. The following sections highlight the major research efforts in each of the four directions.

### 2.1. Accuracy 

Most proposals that focus on improving the accuracy of vehicular localization rely on cooperative positioning. The objective of cooperative positioning is to utilize the network localization resources by allowing neighboring nodes to work together to cooperatively improve the accuracy of their location via the periodic exchange of location information. The focus of research in this area is centered on three main themes: integrating ego measurements with measurements sent by neighbors [2,6,7], assessment of neighbors’ location measurements by analyzing the communication message characteristics [4,8,9,10], and integrating ego or neighbor measurements with map information [3]. Cooperative schemes were proposed to fuse data pertaining to multiple sensors within the vehicle together with data received from other neighbor vehicles in order to obtain relative position estimates [6]. Cameras were used as sensors that provide relative distance measurements [2], and those measurements were fused with the location information exchanged among vehicles. Some schemes incorporate only GPS information to compute inter-vehicle distances for cooperative localization [7]. Local topology information was generated using GPS and ranging sensors, and then exchanged among vehicles [4]. Recently, the characteristics of the Dedicated Short-Range Communications (DSRC) standard were used to enhance the GPS position estimates for vehicles [8]. The concept of centroid localization was expanded by assigning weights to vehicles’ positions based on the Signal-to-Interference-plus-Noise Ratio (SINR) values of exchanged DSRC messages [9]. The Carrier-to-Noise (CNR) ratio was used to mitigate the errors in exchanged GPS pseudorange measurements in order to improve the accuracy of distance ranging [10]. Map matching was also used to verify the accuracy of position estimates produced by Particle filters that fuse GNSS and odometer measurements with the Time-of-Arrival of exchanged DSRC packets [3]. Each vehicle can then merge the different topologies received from neighbors in order to obtain more accurate position estimates. Augmentation systems such as EGNOSS/EDAS were used to mitigate the errors in the Global Navigation Satellite System (GNSS) absolute positioning, while car-to-car communication coupled with group map matching were used to provide relative positioning [11].

### 2.2. Data Fusion

Data fusion is used for vehicle localization both in the autonomous [12,13] and cooperative modes [6]. While GPS measurements are considered the primary source of location information, additional sources of information, such as on-board sensors and cameras as well as digital maps, were used to compensate for GPS errors [12]. The use of different flavors of Kalman filters for location estimation via the fusion of vehicle sensors and GPS measurements was investigated [13].

Landmarks and map information were used as auxiliary information sources to be fused with a vehicle’s own motion measurements. Vehicles can communicate with fixed landmarks that are assumed to be integrated into the road infrastructure in order to measure the Angle-of-Arrival and the Round-Trip Time-of-Flight [14]. Vehicles then couple this information with their own movement dynamics in order to produce an extended Kalman filter that estimates the vehicle’s position. A Monte Carlo localization scheme was adopted for vehicle position estimation via the fusion of LIDAR and vehicle odometry measurements [15]. LIDAR (Light Detection and Ranging) measurements were used to provide an abstraction of the environment in order to account for the constrained vehicle movement. Map and lane abstractions were also used for vehicle localization, with vision sensors used to extract the lane markings [16].

Fusion of different localization techniques has been proposed in order to improve localization accuracy and provide reliable quantification of the accuracy of estimates made by different sources [17]. The authors claim that having enough knowledge about several localization techniques can enable the system to choose the best suitable technique or ensemble techniques for the current time, situation, application and objective.

### 2.3. Modeling of Measurement Errors

Traditionally, the localization measurement errors are modeled as Gaussian distribution random variables. However, several proposals in the literature addressed the problem of modeling localization error to provide better models in order to capture the measurement error behavior. Most of the work addressing this problem can be categorized into two categories: theoretical, as in the proposal in [18], which models the localization error by a uniform random variable distribution; and empirical, as in [19], which uses Received Signal Strength Indicators (RSSI) to build a Time of Arrival ranging error model. Simulation studies have also been used to analyze localization error in [20]. The aforementioned schemes focus on modeling the measurement error. However, no proposals addressed if the measurement errors in successive measurements over time are correlated or not, and the time correlation among the measurement error time series has not been modeled.

### 2.4. Benchmarking

There is little work that is focused on the benchmarking of measurement errors. The authors in [1] use the International Organization for Standardization Guide to the Expression of Uncertainty in Measurement as a reference to their proposed measurement standard for automotive vehicle localization and its measurement uncertainty evaluation. The objective of their work is to provide a benchmarking tool for evaluating the accuracy and availability of localization systems in the market to provide assurance of safety of these localization schemes.

Even with the proliferation of cooperative positioning proposals, they still fall behind the accuracy requirements of safety-oriented vehicular applications. Vehicular safety applications, such as collision avoidance and lane-level navigation, need sub-meter positioning accuracy [21]. In addition, cooperative positioning depends on message exchange, which can only work well in dense traffic scenarios [22]. None of the current research work investigates the time correlation between successive measurement errors. Motivated by this observation, in the following section we proceed to investigate the existence of such time correlation, and we propose a prediction model that incorporates this correlation in order to enhance the prediction accuracy of location information.

## 3. Mathematical Model 

The proposed mathematical model incorporates three main steps: establishing the existence of correlation for a time series of consecutive position measurements, computing the degree of correlation, and defining a model for positioning errors that incorporates correlation of successive measurements. In the following sections we detail each of the aforementioned steps.

### 3.1. Time Correlation of Measurement Error Time Series

The autocorrelation of a random stationary process describes the correlation between values of the process at different times as a function of the time lag between the two times. Since a mobile node’s location is defined over time as it moves, the resulting measurements form a time series. In order to show that time correlation exists among the time series elements, we use the Ljung–Box test [23]. The Ljung–Box test examines whether a group of autocorrelations of a time series are not zero by testing the overall randomness based on multiple lags. The null hypothesis in this test is that the first *m* autocorrelations are jointly zero.

The autocorrelation between times *s* and *t* is defined as:

(1) $$R_{s,t}=\frac{E\left[\left(X_{t}-{\text{μ}}_{t}\right)\left(X_{s}-{\text{μ}}_{s}\right)\right]}{{\text{σ}}_{t}{\text{σ}}_{s}}$$

If *R* is well-defined, its value must be in the range [–1, 1], with 1 indicating perfect correlation and –1 indicating perfect anti-correlation.

When $X_{t}$ is stationary, then autocorrelation depends only on the lag between *t* and *s*. Therefore, the autocorrelation can be expressed as a function of the time lag:

(2) $$R_{\tau }=\frac{E\left[\left(X_{t}-\text{μ}\right)\left(X_{t+\tau }-\text{μ}\right)\right]}{{\text{σ}}^{2}}$$

which is an even function that can be stated as $R_{\tau }=R_{-\tau }$.

The Ljung–Box test is applied to the location measurements corresponding to two datasets corresponding to the traces of two vehicles chosen at random from the TAPASCologne mobility trace [24] and denoted as $v_{1}$ and $v_{2}$ for convenience. The null hypothesis was rejected for both sets of measurements at multiple increasing time lags, with the hypothesis value *h* being equal to 1, indicating perfect time correlation. For the location measurements of both vehicles, *τ*, the correlation time is found to be ≈4 min.

**Figure 1:**
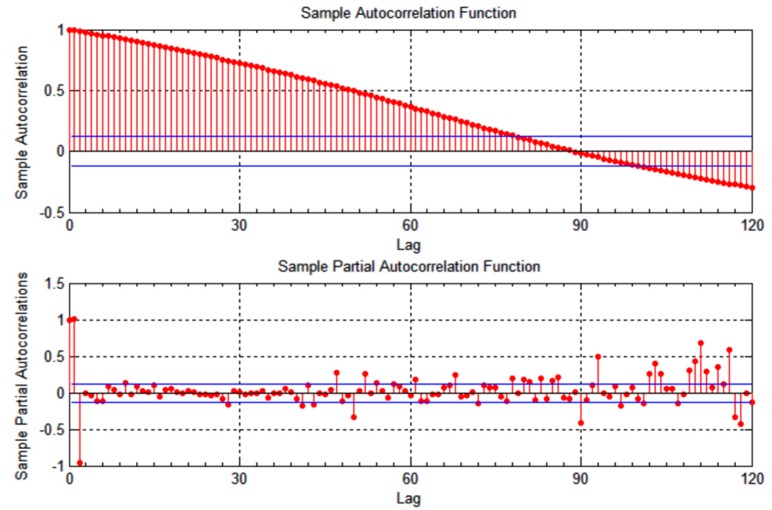
Autocorrelation of longitudinal location measurements for vehicle *v*_1_.

**Figure 2:**
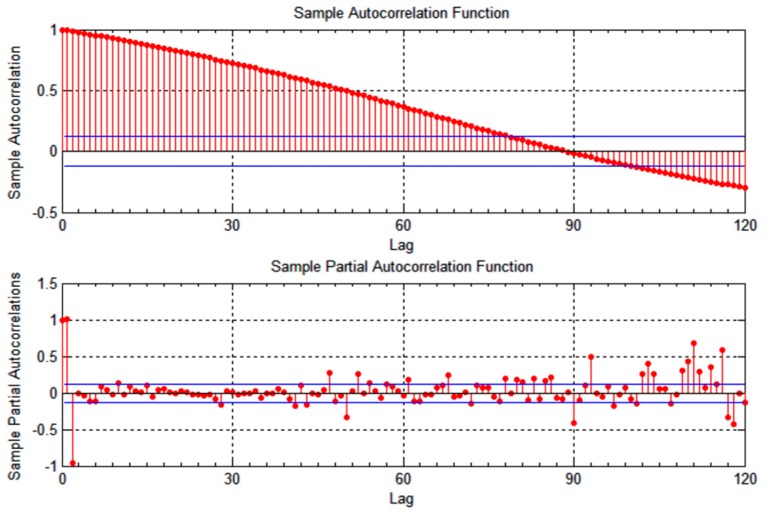
Autocorrelation of latitudinal location measurements for vehicle *v*_1_.

**Figure 3:**
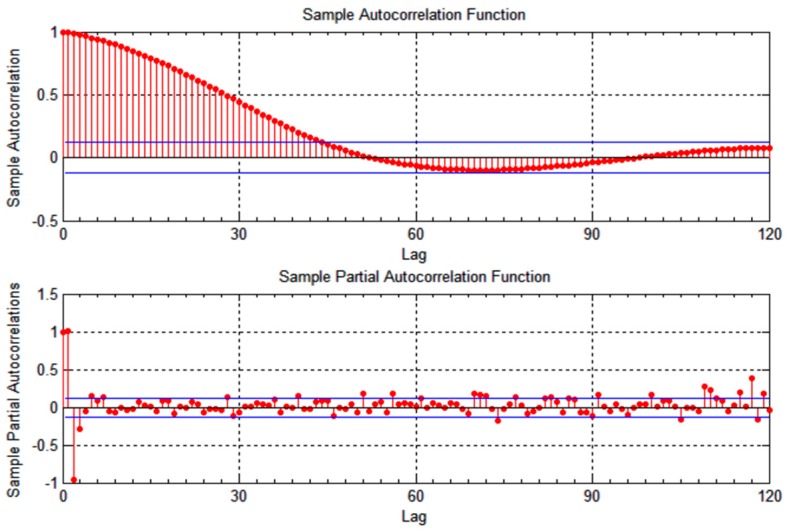
Autocorrelation of longitudinal location measurements for vehicle *v*_2_.

**Figure 4:**
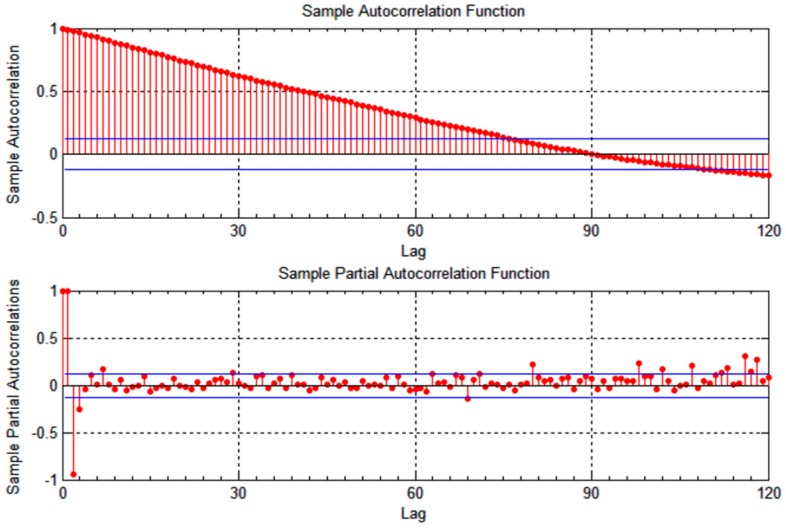
Autocorrelation of latitudinal location measurements for vehicle *v*_2_.

To find out the time window of autocorrelation, beyond which successive location measurements are no longer correlated, we observed when the autocorrelation values approaches zero. Autocorrelation of measurements starts to fade after approximately two minutes for the location measurements of vehicle $v_{1}$, as shown in Figure 1 and Figure 2. The correlation time is slightly lower for vehicle $v_{2}$, becoming less than a minute for the longitudinal measurements, as illustrated in Figure 3 and Figure 4. As the figures show, the location measurements are correlated over a relatively long time epochs. This observation can be attributed to the constrained mobility of vehicles on predictable tracks, with either longitudinal or latitudinal measurements changing slowly as the vehicle moves.

### 3.2. Gauss–Markov Model 

Motivated by the findings in the previous section, we propose a *p*-order Gauss–Markov autoregression model [25] that predicts the future position of a mobile node from its previous mobility trace. We use the Yule Walker equations [26] to determine the degree of correlation between a node’s future position and its past positions.

The *p*-order Gauss–Markov process is a random process that models the randomness of a practical system as a function of time. The order *p* reflects the contribution of the history of the practical system measurements to the current measurement under investigation. For vehicle positioning, we can assume that vehicle movement, and hence its successive positions, follow a similar process, where a vehicle *v*’s future position $X_{n}$ is a time function of *v*’s previous positions. This assumption is reasonable because a vehicle’s movement is constrained by the road map and its movement dynamics. A *p*-order Gauss–Markov process is defined as:

(3) $$X_{n}=A_{1}X_{n-1}+A_{2}X_{n-2}+:::+A_{p}X_{n-p}+\emptyset_{n}$$

where n = 1, 2, …, N is the discrete time interval of measurements, $A_{i},\;i\leq p$, are model parameters, *p* is the number of parameters in the model, and $\emptyset_{n}$ represents the independent white Gaussian. The goal is to predict $X_{n}$ based on the linear combination of $X_{n-1},\;X_{n-2},\;\ldots X_{n-p}$ by estimating the parameters $A_{i}$, and the variance of $\emptyset_{n},$ $\sigma_{\emptyset }^{2}$, hence, fully characterize the model. The best linear prediction of $\tilde{X_{n}}$ with minimum mean square error is the one that meets two criterion; $\tilde{X_{n}}$ can be defined as a linear combination of $X_{n-1},\;X_{n-2},\;\ldots X_{n-p}$ and the prediction error, $\emptyset_{n}=X_{n}-\tilde{X_{n}}$ is uncorrelated with the linear combination of $X_{n-1},\;X_{n-2},\;\ldots X_{n-p}$. 

### 3.3. Calculating the p-Order Gauss–Markov Model Parameters

#### 3.3.1. Calculating the Order *p*

$X_{n}$ is a stationary process, and is considered autoregressive of an order *p*. There exist several methods to estimate the best order *p* of an autoregressive model such as the Akaike information criterion (AIC), Akaike Final Prediction Error (FPE), Minimum Description Length (MDL), and the reflection coefficients of a Yule Walker method. We chose to employ AIC method to estimate the best order of the autoregressive model. AIC is used to compare different statistical models as a measure of their relative quality for a given set of data.

(4) AIC = 2p – 2ln(L)

where *L* is the maximum likelihood of the model given the set of data, and reflects the goodness of fit of the model; *i.e.*, smaller values indicate worse fit. AIC is based on balancing the goodness of fit of a model with the complexity of the model; *i.e.*, the order *p*. In fact, increasing the order of a model does not necessarily provide a more accurate estimation. The AIC method, while giving preference to models with a better goodness of fit, it simultaneously penalizes models with higher order *p*, to avoid settling to models with larger *p* and less gain in the goodness of fit. In a nutshell, AIC provides a tradeoff between accuracy and complexity. 

**Figure 5:**
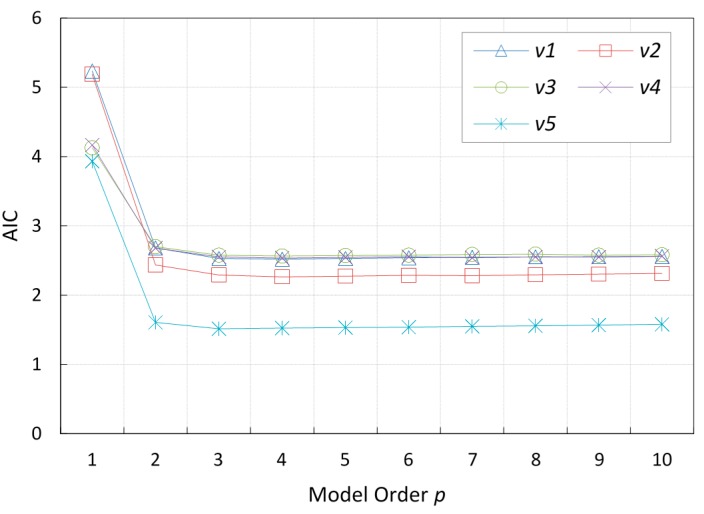
*AIC* values for different *AR* models, compared for five vehicle trips.

For a set of data and a set of autoregressive models with different values of *p*, the best model is identified as the model that has the minimum AIC value. Figure 5 shows the AIC values for different autoregressive models with different orders, calculated for five different vehicles whose location information were extracted from the TAPASCologne vehicular mobility trace [24]. We can observe that the lowest AIC value for the majority of the vehicles is achieved when the autoregressive model order *p* is equal to 4, and that the model achieves an optimal balance between prediction accuracy and model complexity when p=2. However, we will show through simulation experiments that the simplest order, at p=1, is sufficient for prediction. The gain in prediction precision that is achieved by an autoregression model of order 4 is not large enough to justify the complexity cost incurred. 

#### 3.3.2. Calculating the Parameters

After estimating the best order *p*, we can estimate the parameters $A_{i},\;i=1,..p$, and once $A_{i}$ is estimated, ${\text{σ}}_{\emptyset }^{2}$ can be estimated in turn. There are several methods to estimate the coefficients of the *p*-order Gauss–Markov model AR(p), such as the Markov Chain Monte Carlo, the least square optimization method, and the method of moments using Yule Walker equations. We chose to use the method of moments through the Yule Walker equations, due to its simplicity and the fact that it produces consistent estimators. Following [26], we provide the estimation of $A_{i\leq p}$ and ${\text{σ}}_{\emptyset }$ as follows:

Referring to Equation (3), we proceed to solve for the parameters of AR(p). Multiplying Equation (3) by $X_{n-p}$, we get:

(5) $$X_{n-p}X_{n}\;=\overset{p}{\underset{i=1}{\sum }}A_{i}X_{n-p}X_{n-i}\;+\;X_{n-p}\emptyset_{n}$$

Taking the expectation of Equation (5), we get:

(6) $$E(X_{n-p}X_{n})\;=E\left(\overset{p}{\underset{i=1}{\sum }}A_{i}X_{n-p}X_{n-i}\;+\;X_{n-p}\emptyset_{n}\right)$$

(7) $$E(X_{n-p}X_{n})\;=\overset{p}{\underset{i=1}{\sum }}A_{i}E\left(X_{n-p}X_{n-i}\right)\;+\;E(X_{n-p}\emptyset_{n})$$

(8) $$E(X_{n-p}\emptyset_{n})=\{\begin{array}{c} 0,\;p\neq 0\\ {\text{σ}}_{\emptyset }^{2},\;p=0\; \end{array}$$

Equation (8) follows from the observation that $X_{n-p\;}\text{and}\;\emptyset_{n}$ are uncorrelated for p≠0, because $\emptyset_{n}$ of the current time is unrelated to ${\text{X}}_{\text{n}-\text{p}}$ and thus uncorrelated with previous values of the process, hence,

(9) $$E(X_{n-p}X_{n})\;=\overset{p}{\underset{i=1}{\sum }}A_{i}E\left(X_{n-p}X_{n-i}\right)\;$$

Dividing Equation (9) by N the observation window of the process ${\text{X}}_{\text{n}}$, and using the fact that the process is stationary, $\text{E}({\text{X}}_{\text{n}-\text{p}}{\text{X}}_{\text{n}})$, is a function of the time lag, *p*, only, and $\text{E}({\text{X}}_{\text{n}-\text{p}}{\text{X}}_{\text{n}})$, can be denoted by $\;{\text{c}}_{\text{p}}$, also using ${\text{c}}_{\text{p}}={\text{c}}_{-\text{p}}$, Equation (9) can be written as

(10) $$c_{p}=\;\overset{p}{\underset{i=1}{\sum }}A_{i}c_{i-p}$$

Dividing $c_{p}$ by $c_{0}$, we get:

(11) $$r_{p}=\;\overset{p}{\underset{i=1}{\sum }}A_{i}r_{i-p}$$

where ${\text{r}}_{\text{p}}$ is the autocorrelation coefficient. Rewriting the equations for all $r_{1\leq i\leq p}$; *i.e.*, $r_{1}=\;\overset{p}{\underset{i=1}{\sum }}A_{i}r_{i-1}$, $r_{2}=\;\overset{p}{\underset{i=1}{\sum }}A_{i}r_{i-2}$, $r_{3}=\;\overset{p}{\underset{i=1}{\sum }}A_{i}r_{i-3}$ …., $r_{p}=\;\overset{p}{\underset{i=1}{\sum }}A_{i}r_{i-p}$ in matrix form, we have:

(12) R=βA

(13) $$R=\left[\;\begin{array}{c} r_{1}\\ \ldots \\ r_{p} \end{array}\right],A=[\;\begin{array}{c} A_{1}\\ \ldots \\ A_{p} \end{array}],and\;\beta =\left[\begin{array}{ccc} r_{0} & r_{1} & \ldots \\ r_{1} & r_{0} & \\ \begin{array}{c} r_{p-1} \end{array} & \begin{array}{c} \vdots \end{array} & \begin{array}{c} r_{1} \end{array} \end{array}\;\begin{array}{c} r_{p-1}\\ r_{p-2}\\ \begin{array}{c} \vdots \\ r_{0} \end{array} \end{array}\right]$$

Note that β is full-rank and symmetric matrix*,* hence it is guaranteed that its inverse exist. Thus, *A* can be estimate from Equation (12) as $\tilde{A}=\beta^{-1}R$.

Once the model parameters are estimated, the noise variance ${\text{σ}}_{\emptyset }^{2}$ can be estimated by setting p=0 in Equation (7):

(14) $$E(X_{n}X_{n})\;=\overset{p}{\underset{i=1}{\sum }}A_{i}E\left(X_{n}X_{n-i}\right)\;+\;E(X_{n}\emptyset_{n})$$

Using the fact that $X_{n}$ is a stationary process again, ${\text{c}}_{i}=\;{\text{c}}_{-i}$, and ${\text{σ}}_{\emptyset }^{2}=E(X_{n}\emptyset_{n})$, Equation (14) can be written as:

(15) $$c_{0}=\;\overset{p}{\underset{i=1}{\sum }}A_{i}c_{i}+{\text{σ}}_{\emptyset }^{2}$$

(16) $${\text{σ}}_{\emptyset }^{2}=c_{0}-\;\overset{p}{\underset{i=1}{\sum }}A_{i}c_{i}$$

## 4. Applications of the Time-Correlated Localization Measurement Errors Model

The proposed time-correlated localization errors model can be beneficial for location prediction when GPS is not available due to variations in urban structure (e.g., entry into tunnels). The model can also improve the localization accuracy because it incorporates the time dependency of measurements into prediction. The model does not need information beyond the initial position measurements of a vehicle. This reduces the complexity of processing in terms of data filtering and fusion. The *p*-order Gauss–Markov model can also be used as a prediction tool that enhances the performance of existing localization schemes, such as the Kalman filter [27]. In the following section, we illustrate through simulations and real vehicle mobility traces how the model can be used standalone as well as a prediction tool for the Kalman filter. 

## 5. Simulation Results and Discussion

### 5.1. Simulation Setup 

The proposed model was evaluated using a two-hour excerpt of the TAPASCologne vehicular mobility trace generated for the city of Cologne, Germany [24]. TAPASCologne is an initiative by the Institute of Transportation Systems at the German Aerospace Center (ITS-DLR), aimed at reproducing realistic car traffic in the greater urban area of the city of Cologne in Germany. Vehicle mobility was generated synthetically using the SUMO microscopic vehicular mobility simulator [28], and traffic demand was generated using the Travel and Activity PAtterns Simulation (TAPAS) methodology. The original trace covers a region of 400 square kilometers of the city of Cologne for a period of 24 h, comprising more than 700,000 individual car trips. This dataset is the most standardized dataset we could find, with detailed location and speed measurements over a time window that captures most of the traffic patterns that can be experienced in a city with urban and suburban areas. The time granularity between vehicular location measurements is one second, which is reasonable in capturing vehicles’ mobility over time. Vehicle speed varied between 0 km/h and 90 km/h, covering most traffic scenarios. The two-hour excerpt was enough to establish autocorrelation and evaluate its effect on location prediction and error measurements.

The two-hour excerpt trace was preprocessed to extract five traces corresponding to five individual vehicle trips. The five individual vehicles are chosen at random using a uniform distribution-based random number generator, and are labeled $v_{1}$ to $v_{5}$ throughout the remainder of the paper for ease of reference. A single vehicle trip consists of a time-stamped sequence of *xy* position coordinates in meters as well as the vehicle’s speed in meters per second. The selected vehicles’ total trip times are 257 s each. The experiments were conducted using Matlab (R2013a). The estimation of the AR(p) model order was performed using the System Identification Toolbox for p = 1:10, with the location information for one vehicle used for cross validation. The AIC values for the estimated model were compared for the five vehicles and the majority of them exhibited a best model fit at p = 4, as illustrated in Figure 5. 

We chose two of the five vehicles and used their location measurements to generate the model parameters and validate its performance. The two vehicles used for model estimation and validation were designated $v_{1}$ and $v_{2}$, respectively. The location measurements for vehicle $v_{1}$ were used to infer the model parameters, and the location measurements for vehicle $v_{2}$ were used to validate the model performance using the parameters generated by $v_{1}$’s measurements. We estimated the unknown model coefficients and noise variance for p = 1, 2, 4 for the location information of both vehicles using the Yule Walker equations.

### 5.2. Simulation Results 

Three sets of experiments were conducted to investigate two modes of operation for the proposed model. First, we investigated the model performance when used as a standalone model for prediction, for example when GPS is not available. Second, we investigated the performance of our model when integrated into a localization algorithm such as a Kalman filter algorithm. The Kalman filter was chosen because of its popularity as a localization scheme, as well as its ease of implementation. Third, we applied the Kalman filter that was adjusted by the Gauss–Markov model parameters to a real-life dataset in order to assess the accuracy of the enhanced localization scheme and rule out overfitting. The first two sets of experiments study the tradeoff between reducing the complexity of the *p*-order Gauss–Markov model and the location prediction accuracy, as well as assess the increase in accuracy of localization when using the model in the prediction step of the Kalman filter. The third experiment was used for validation of the model. Each model is designated AR(p) for ease of reference.

**Figure 6:**
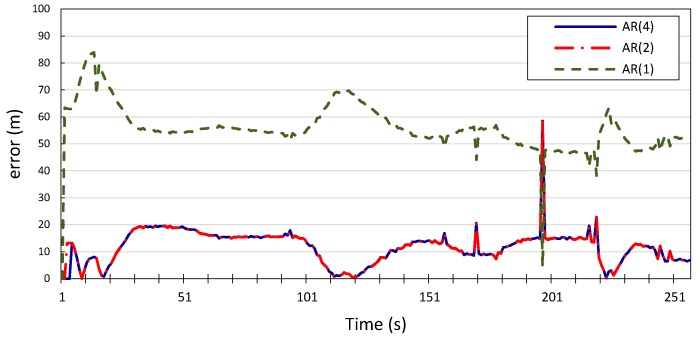
Longitudinal position error for vehicle *v*_1_ using autoregression model with *p* = 1, 2, 4.

**Figure 7:**
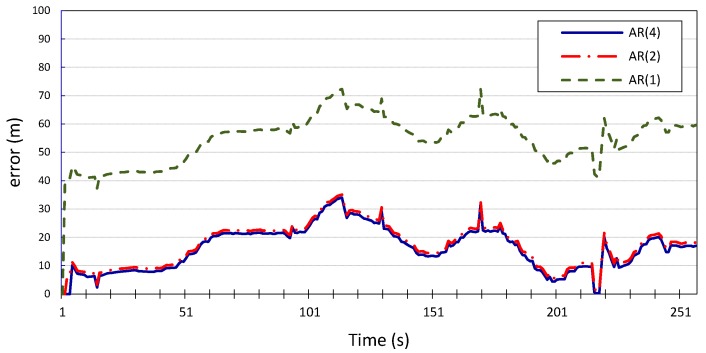
Latitudinal position error for vehicle *v*_1_ using autoregression model with *p* = 1, 2, 4.

**Figure 8:**
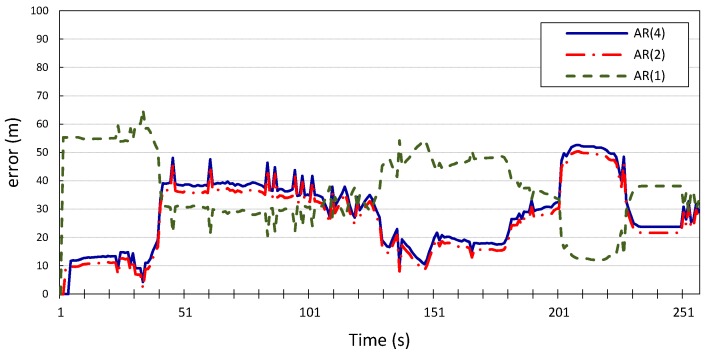
Longitudinal position error for vehicle *v*_2_ using autoregression model with *p* = 1, 2, 4.

**Figure 9:**
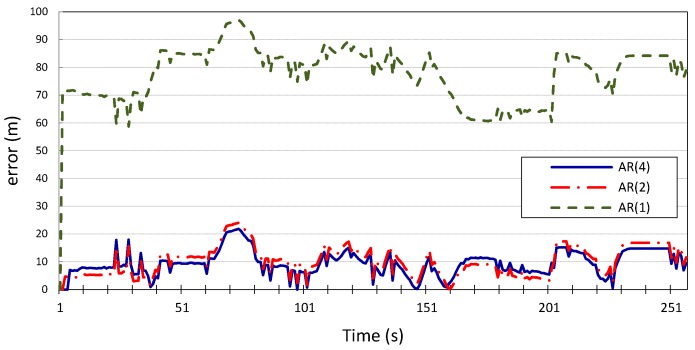
Latitudinal position error for vehicle *v*_2_ using autoregression model with *p* = 1, 2, 4.

The model parameters $A_{i}$ that were produced by the Yule Walker equations for the two mobility traces for vehicles $v_{1}$ and $v_{2}$ are listed in Table 1. For the first set of experiments (standalone prediction), the results in Figure 6 and Figure 7 show the errors in the longitudinal (*x*) and latitudinal (*y*) position estimates made by the Gauss–Markov model for p=1, 2, 4 for vehicle $v_{1}$. The figures show that higher model orders correspond to reduced estimation error of the future location coordinates of the vehicle; p=2 and p=4 can predict the vehicle’s location with an average error of 11 m for *x* and 16 m for *y*, with inconsiderable improvement made by the model with p=4 compared to the model with p=2. However, when p=1, the average error is 55 m for both *x* and *y*. This is further corroborated by the results produced when applying the same Gauss–Markov model on the location measurements of vehicle $v_{2}$, which has been designated for validation, as shown in Figure 8 and Figure 9. We can conclude that p=2 is a reasonable compromise between complexity and accuracy and therefore is sufficient for depolyment of the error model as a standalone prediction tool. The tradeoff performance provided by the model when p=2 will make the model’s prediction robust even when used in fast-fading channel scenarios, which may exhibit lower correlation windows, since the correlation window in this case is small enough to allow for correlation to be manifested, while still producing accurate prediction. This makes the model applicable in different scenarios with potentially varying correlation windows.

**Table 1:** Autoregression model parameters for the two vehicle mobility traces used for experiments.

Vehicle Traces	Model	Longitude				Latitude			
		*A*_1_	*A*_2_	*A*_3_	*A*_4_	*A*_1_	*A*_2_	*A*_3_	*A*_4_
*trace* *v*_1_	*AR*(1)	0.996				0.9961			
	*AR*(2)	0.9978	0.0018			0.9977	0.0016		
	*AR*(4)	0.9978	0	0	0.0018	0.9977	0.0001	0	0.0016
*trace* *v*_2_	*AR*(1)	0.9963				0.996			
	*AR*(2)	0.9992	0.0029			0.9976	0.0016		
	*AR*(4)	0.9992	0.0001	0.0002	0.0028	0.9976	0.0001	0.0001	0.0015

For the second experiment, we want to investigate the integration of the model into a localization algorithm. The Gauss–Markov model was integrated into the Kalman filter localization scheme proposed in [29] in order to illustrate how error modeling and parameter estimation enhance the accuracy of localization. We will experiment with p=1, 2, 4 to assess whether reducing the model complexity using p=1 will provide comparable results to higher orders.

The Kalman filter involves two steps: a prediction step and a measurement update step. The prediction step assumes that the state of a system at time *t* evolved from the prior state at t−1 according to:

(17) $$x_{t}=F_{t}x_{t-1}+B_{t}u_{t}+\emptyset_{t}$$

where $x_{t}$ is the state vector containing system information at time *t*, $u_{t}$ is a vector containing control inputs, $F_{t}$ is a state transition matrix that applies the effects of the system state at t−1 to its state at time *t*, *B_t_* is a control input matrix that serves a similar purpose to *F_t_* but for the control inputs, and $\emptyset_{t}$ is the process noise. In our experiments, the transition matrix $F_{t}$ was composed using the parameter values $A_{p}$ that were produced by the Yule Walker equations, which are listed in Table 1. A different matrix was constructed for each model using the longitude and latitude parameter values. The size of the transformation matrix was the same size of the dataset. The control matrix $B_{t}$ was set to zero since there are no control inputs. The measurement step performs corrections to the predicted location, according to the equation:

(18) $$z_{t}=H_{t}x_{t}+w_{t}$$

where $z_{t}$ is the vector of measurements, $H_{t}$ is a transformation matrix which is set to default value of 1 in our experiments, and $w_{t}$ represents the measurement noise.

**Figure 10:**
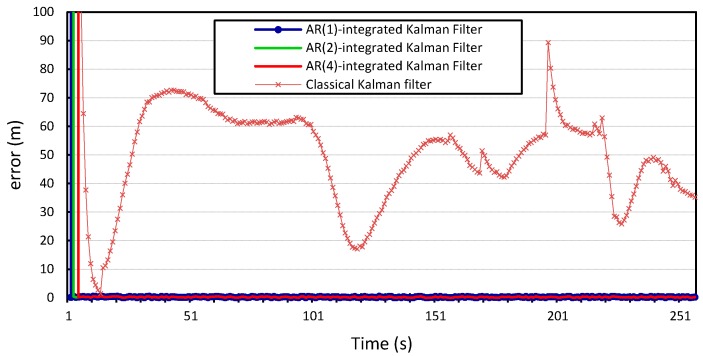
Longitudinal positioning error produced by the standard Kalman filter and the *Autoregression*-Kalman filter for vehicle $v_{1}$.

**Figure 11:**
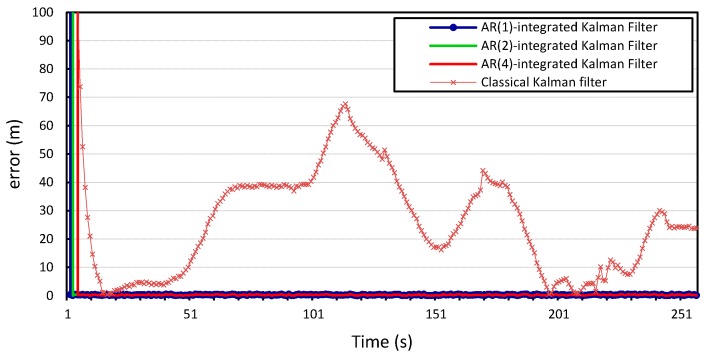
Latitudinal positioning error produced by the standard Kalman filter and the *Autoregression*-Kalman filter for vehicle $v_{1}$.

**Figure 12:**
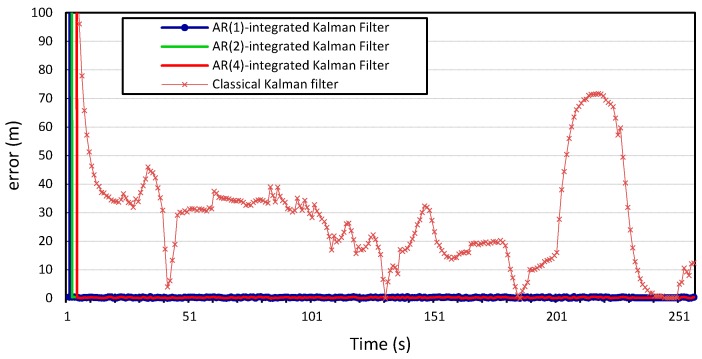
Longitudinal positioning error produced by the standard Kalman filter and the *Autoregression*-Kalman filter for vehicle $v_{2}$.

**Figure 13:**
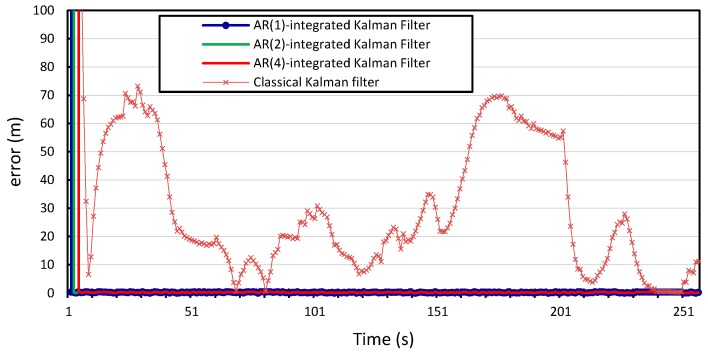
Latitudinal positioning error produced by the standard Kalman filter and the *Autoregression*-Kalman filter for vehicle $v_{2}$.

The process and measurement noise in Kalman filter-based localization algorithms are usually assumed to be zero-mean Gaussian white noise. Our proposed error model can compute the noise variance in location information. Therefore, we incorporate it into the prediction step of the Kalman filter and study how this will improve the localization algorithm. The AR-integrated Kalman filter is then applied to the two time series location information of vehicles $v_{1}$ and $v_{2}$, and the results shown in Figure 10, Figure 11, Figure 12 and Figure 13 compare the localization error for the three error models; p=1, p=2, and p=4. We note that the computational complexity of the Kalman filter and the *autoregression*-integrated Kalman filter is dominated by the matrix multiplication, and therefore grows as $O\left(n^{2}\right)$, where n is the number of location measurements. We also note that the computational complexity of the AR-integrated Kalman filter implementation and the standard Kalman filter implementation are identical. This is because the integration of the AR model into the Kalman filter involves the replacement of the transition and error matrices and the extension of prediction step to accommodate computations when p=2 and p=4.

**Figure 14:**
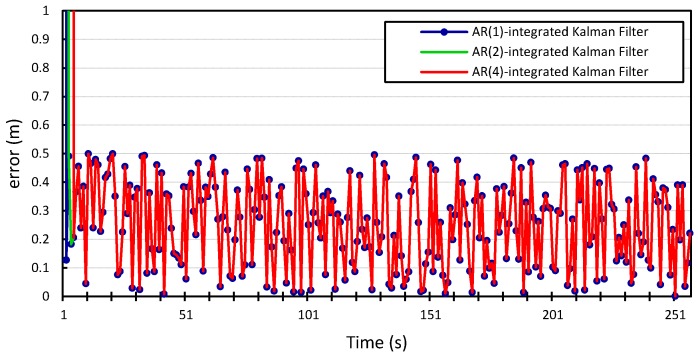
Zoom in on the longitudinal positioning error produced by the *Autoregression*-Kalman filter for vehicle $v_{1}$.

**Figure 15:**
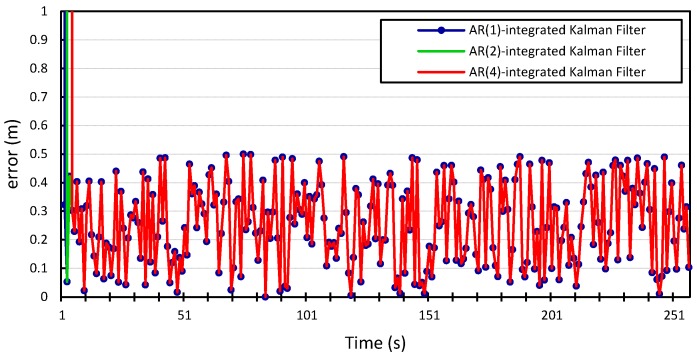
Zoom in on the latitudinal positioning error produced by the *Autoregression*-Kalman filter for vehicle $v_{1}$.

We can see that incorporating our error model into the Kalman filter outperforms the results produced by the Kalman filter with zero Gaussian error, which we call the standard Kalman filter for convenience. Similar accuracy gains are obtained when the error model is integrated into the Kalman filter and applied to the location measurements of vehicle $v_{2}$ whose location information is used for validation. We can also observe that there is no observable gain in accuracy when using a higher Gauss–Markov order, since p=1 provides almost identical results to p=4. The average measurement error for the standard Kalman filter applied to the location measurements of vehicle $v_{1}$ is 56 m and 29 m for *x* and *y*, respectively, compared to 0.25 m produced by the Kalman filter that is integrated with the autoregression model of order 1. The Kalman filter that was integrated with the autoregression model of order 4 has an average measurement error of 0.24 m, which is very close to that produced by the autoregression model of order 1. This can be further illustrated in Figure 14 and Figure 15, which provide a more detailed view of the behavior of the model for p=1, 2, 4. The fluctuations in error measurements are due to the constant variability in the vehicles’ position measurements, as was found upon further inspection of the original time-series for longitudinal and latitudinal location measurements of both vehicles.

#### 5.3. Verification of Model

**Figure 16:**
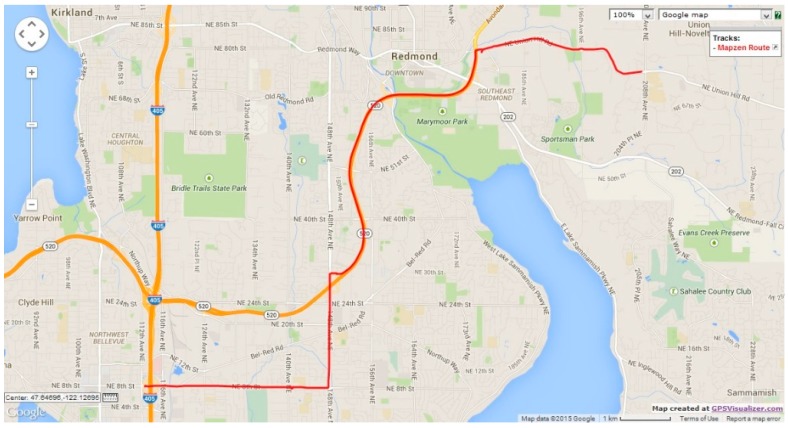
OpenStreetMap route for verification dataset 1.

**Figure 17:**
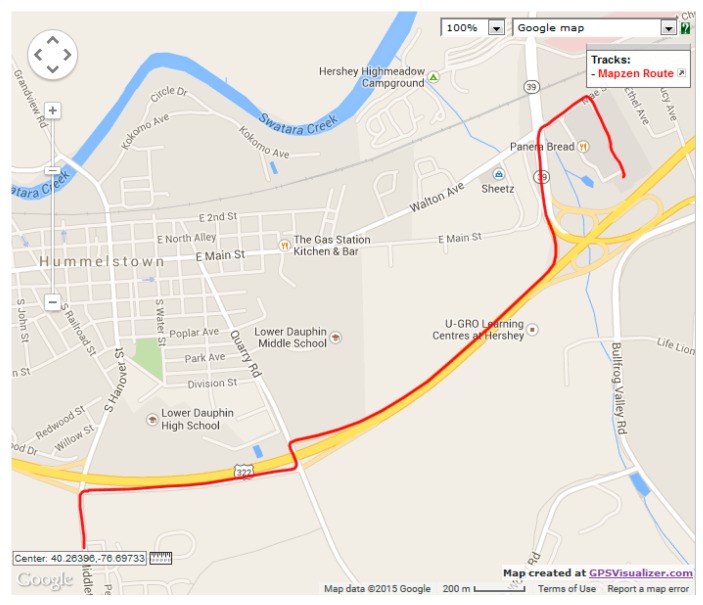
OpenStreetMap route for verification dataset 2.

**Figure 18:**
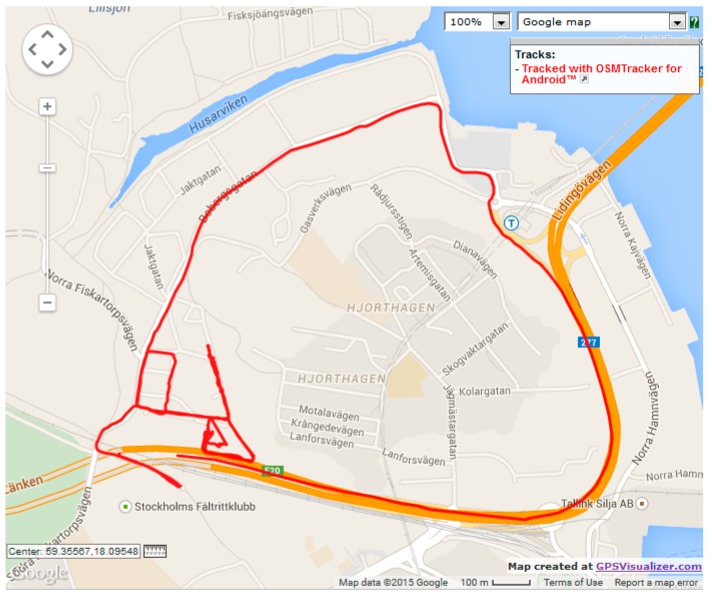
OpenStreetMap route for verification dataset 3.

In order to verify our AR model, we tested the model against three realistic datasets. We chose three vehicular mobility traces from OpenStreetMap [30]. The first trace (dataset 1), which is illustrated in Figure 16, represents a single vehicle trip along the route between Northeast Union Hill Road and Bravern 1, Bellevue, WA. The total trip time was 30 min and 45 s (totaling 1845 data points), and vehicle speed ranged from 0 km/h to 104.4 km/h. The second trace (dataset 2), illustrated in Figure 17, represents a single vehicle trip between East Main Street and the Gelder Park, Derry, PA, USA. The total trip time was 5 min and 45 s (totaling 343 data points), and speed ranged from 0 km/h to 96.7 km/h. The third trace (dataset 3), illustrated in Figure 18, depicts a vehicle’s trip around a new residential area in Stockholm, Sweden. The total vehicle trip time was 25 min and 56 s (totaling 1313 data points). There is no mention of the vehicle speeds for the third dataset. For the three datasets, location measurements were taken every second. 

**Figure 19:**
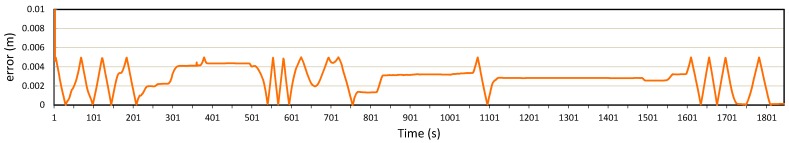
Longitudinal positioning error produced by *Autoregression*-Kalman filter for *dataset* 1.

**Figure 20:**
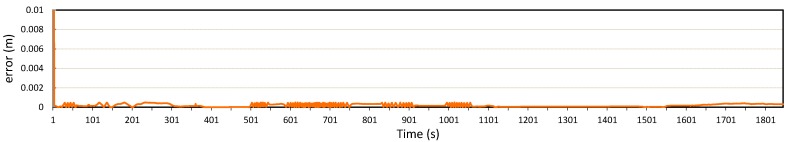
Latitudinal positioning error produced by *Autoregression*-Kalman filter for *dataset* 1.

**Figure 21:**
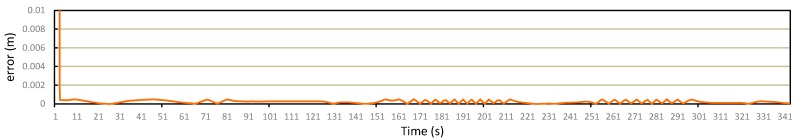
Longitudinal positioning error produced by *Autoregression*-Kalman filter for *dataset* 2.

**Figure 22:**
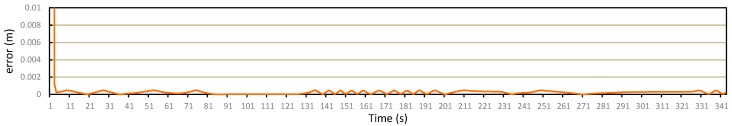
Latitudinal positioning error produced by *Autoregression*-Kalman filter for *dataset* 2.

**Figure 23:**
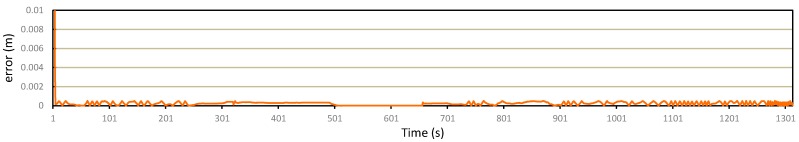
Longitudinal positioning error produced by *Autoregression*-Kalman filter for *dataset* 3.

**Figure 24:**
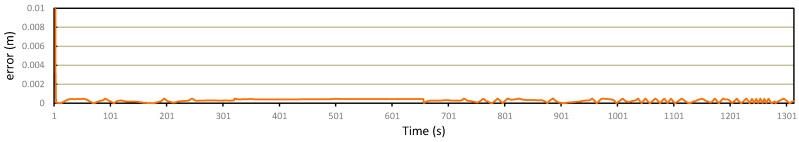
Latitudinal positioning error produced by *Autoregression*-Kalman filter for *dataset* 3.

As with the previous experiment set, the model parameters and process noise were extracted from each of the datasets using the autoregression model of order 1. The Kalman filter was integrated with the model parameters and applied to the location measurements. Figure 19, Figure 20, Figure 21, Figure 22, Figure 23 and Figure 24 show, respectively, the longitudinal and latitudinal position measurement errors produced by the Kalman filter when the Gauss–Markov model is used for the prediction step when applied to the OpenStreetMap traces. The margin of error for all three datasets stays within a very small and negligible range (0.005 m), which indicates the high level of accuracy produced by the localization model. The larger variation in longitudinal error in comparison to the latitudinal error in dataset 1 is due to the larger variation rate in longitudinal measurements compared to a small overall change in the latitudinal measurements. 

The localization model performs well regardless of the road scenario—highway, residential, or a combination of both. Error variation is miminal at the intervals during which the vehicles do not change their positions, as can be noticed in the interval between the seconds 1101 and 1550 in dataset 1 and between the seconds 310 and 650 for dataset 3, for example. We notice that error in the third section of the position measurements for dataset 3 exhibits more fluctuations. When the corresponding vehicle’s route is closely inspected, it can be evident that this variation is due to the rather unsmooth movement along some sections of the residential area. We can assume that this error variation can be controlled if vehicle speed is incorporated into the prediction model, which is part of our future work.

## 6. Conclusions and Future Work

Vehicular localization accuracy remains a key issue for vehicular safety applications. In this paper, we proposed the use of autoregressive *p*-order Gauss–Markov model to model successive location measurement errors and incorporate these measurement errors in the prediction of a vehicle’s future position given its past position measurements. We used our model as a prediction step in the Kalman filter localization scheme, and results showed substantial accuracy gains over a standard Kalman filter at the same level of computation complexity. We could infer the autocorrelation time between location measurements, which can be used in the future to enhance the accuracy of localization algorithms. We intend to incorporate diverse measurement types into the model in order to further enhance its accuracy, and provide means for estimators to define upper bounds on the error measurements of location estimates.
